# Comments on: Chari, R.; Burke, T.A.; White, R.H.; Fox, M.A. Integrating Susceptibility into Environmental Policy: An Analysis of the National Ambient Air Quality Standard for Lead. *Int. J. Environ. Res. Public Health* 2012, *9*, 1077–1096

**DOI:** 10.3390/ijerph10020742

**Published:** 2013-02-21

**Authors:** Teresa S. Bowers

**Affiliations:** Gradient, 20 University Road, Cambridge, MA 02138, USA; E-Mail: tbowers@gradientcorp.com; Tel.: +1-617-395-5000.

**Keywords:** lead, blood lead, IQ, socio-economic status, National Ambient Air Quality Standard

## Abstract

A recent publication in *International Journal of Environmental Research and Public Health* by Chari *et al.* [1] provides no basis for changing the National Ambient Air Quality Standard (NAAQS) for lead in the U.S. to protect children in low socio-economic (SES) populations. The studies selected by Chari *et al.* for analysis do not provide comparable information on regression coefficients for the blood lead level-IQ relationship. The coefficients differ from one another more on the basis of unequal blood lead metrics, ages of blood lead measurement, and differences in covariate adjustments and standardization, than the difference postulated by Chari *et al.* to correspond to low *vs.* high SES populations.

In [1], Chari *et al.* examine literature that summarizes the relationship between blood lead levels in young children and cognitive deficits (IQ) for high and low socio-economic status (SES) populations. Although it has long been observed that there is an inverse association between SES and blood lead levels, the authors go beyond this observation to hypothesize that differences in the dose-response relationship between blood lead and IQ exist depending on the SES status of the population. As a result, they conclude that EPA’s decision framework for development of the National Ambient Air Quality Standard (NAAQS) for lead should result in a lower NAAQS in order to protect these susceptible populations. However, Chari *et al.*’s analysis is not adequate for this purpose.

Chari *et al.* rely on four publications that examine the blood lead-IQ relationship as a function of SES status: Dietrich *et al.* [2]; McMichael *et al.* [3]; Tong *et al.* [4]; and Harvey *et al.* [5]. The ages of the children at the time of blood lead measurement, the blood lead metric, and the type of cognitive test, all differ in these four studies. Dietrich *et al.* and McMichael *et al.* employed the Mental Development Index (MDI) of the Bayley Scales of Infant Development, administered at 6 months by Dietrich *et al.* and at 2 years by McMichael *et al.* Harvey employed the British Ability Scales, administered at 2 years, and Tong *et al.* employed the Wechsler Intelligence Scale for Children (WISC-R), administered at 11–13 years. IQ testing at these various ages obviously takes different forms, and after control for confounders, there is little correlation between the MDI and IQ administered at later ages (Martin *et al.* [6]).

Children’s ages at the time of blood lead measurement range from 10 days (Dietrich *et al.*) to 11–13 years (Tong *et al.*), while the blood lead metric includes a measure of early childhood (Dietrich *et al.*), lifetime average (McMichael *et al.* and Tong *et al.*), and age 2.5 years, approximately peak blood lead (Harvey *et al.*). Further, the McMichael *et al.* and Tong *et al.* studies are of the same Port Pirie children, but at different ages. Blood lead levels are a function of age, peaking at approximately age 2–3 years, and declining into adolescence. Regression coefficients describing the relationship between blood lead level and IQ vary as a function of the age of blood lead measurement and the blood lead metric. This can be seen in Lanphear *et al.* [7] where four blood lead metrics at different ages are separately regressed against IQ measurements, with differing results (see Table 4 in Lanphear *et al.*). The difference in coefficients in the McMichael *et al.* and Tong *et al.* studies may result in part from the age difference. Comparisons of different SES groups must be based on regression coefficients for the same blood lead metric and approximately the same age, or adjusted in some way for the impact of variable blood lead metrics and ages at time of measurement. Although Chari *et al.* note that the variable blood lead metrics are a limitation of their analysis, they don’t indicate the direction of bias associated with this limitation and the subsequent impact on their conclusions. The difference in coefficients proposed by Chari *et al.* for high and low SES populations is similar to the difference seen between studies of children at different ages, suggesting if this bias were considered that there might be no difference in dose-response relationships at different SES levels.

Blood lead levels reported in the Dietrich *et al.* study are hematocrit-adjusted. This adjustment would also have an impact on the magnitude of the regression coefficient, and complicate the comparison of this result to those of other studies.

Additional uncertainty in the Chari *et al.* analysis comes from the authors’ extrapolation of the log-linear regression coefficients to a blood lead level of 2 µg/dL, well below the average blood lead levels in each of the studies. Although extrapolation of linear coefficients to lower blood lead levels may produce a bias towards underestimating effects, extrapolation of log-linear coefficients may produce a bias in either direction. As an example of the impact on the dose-response slope resulting from this extrapolation, Chari *et al.* extrapolate and linearize the regression coefficient from Dietrich *et al.* at 2 µg/dL to be −2.35. However, a later publication on the same data set by Dietrich *et al.* [8] reports a linear decrease of 0.73 MDI points per 1 µg/dL increase in (hematocrit-corrected) blood lead level across the blood lead range in the study. The difference between coefficients of −0.73 and −2.35, which are derived in different ways for the same data set, is more than the difference postulated by Chari *et al.* for high *vs.* low SES children, suggesting again that there might be no difference in dose-response relationships at different SES levels.

Complicating the comparison of coefficients among these four studies further, some of the coefficients are adjusted for covariates while others are not, and some are standardized while others are not. The regression coefficients from the McMichael study are unadjusted, while those from the other three studies are adjusted. This difference matters: Bellinger [9] notes that “…adjusting for SES and related covariates can result in reductions of 50% or more in the magnitude of lead’s regression coefficient”. Again, Chari *et al.* should have indicated the direction of this bias. Further, Harvey *et al.* reported a standardized regression coefficient. The value of a standardized coefficient, which can vary from negative to positive one, is not comparable to an unstandardized coefficient. Although Chari *et al.* acknowledge that some coefficients are adjusted and others are not, they neither note the direction of bias associated with this limitation, nor note the lack of comparability with the Harvey *et al.* standardized regression coefficient.

Although the comparison across studies is complicated by the various types of regression coefficients, and the mixed ages, blood lead metrics and cognitive tests, it remains worth reviewing the results found within a study between the high and low SES categories. Chari *et al.* cite two studies that give this comparison: McMichael *et al.* and Tong *et al.* As noted above, these two studies are of the same children, and it is perhaps not surprising that a similar result is found. However, in neither study did the authors find a statistically significant impact of SES on the blood lead-IQ relationship.

The study by Harvey *et al.* also did not find statistical significance. Before adjustment for covariates, Harvey *et al.* reported a correlation between blood lead and IQ that was larger and negative for the manual labor class. Harvey *et al.* [5] present results only for the group of children at age 2.5 years. In a later publication, Harvey *et al.* [10] report the results of a similar analysis done for a group of children at age 5.5 years. These are not the same children, but rather two groups of children at different ages that were selected and studied at approximately the same time. However, in examining the older children, Harvey *et al.* [10] found the opposite result in that the children of fathers engaged in non-manual labor had the higher correlation between blood lead and IQ, with a more negative coefficient, although again unadjusted for covariates. In both studies, Harvey and co-authors found that adjustment of the regression for other predictors of IQ resulted in no statistically significant relationship between blood lead and IQ within the studied social class. Chari *et al.* included the younger children from the first study where the father is engaged in manual labor as low SES in their analysis, but did not include the older children from the second study where the result was the opposite.

Only the study by Dietrich *et al.* found statistical significance, in an analysis of the Cincinnati cohort based on blood lead levels at age of ten days. However Dietrich *et al.* [8] also examined the blood lead levels at age 3 months for this same subset of children with SES less than the median, and found that the relationship between blood lead and MDI was not statistically significant. Chari *et al.* also note that an SES effect was not seen in the Cincinnati cohort in later years.

In summary, the differences in regression coefficients describing the blood lead-IQ relationship between low and high SES populations as put forward by Chari *et al.* are similar to or less than the differences between regression coefficients expected as a result of mixing blood lead metrics, children’s ages, adjusting or not adjusting for covariates, and comparing standardized and non-standardized regression coefficients. Further, the studies selected by Chari *et al.* show little or no statistical significance of an SES effect on the relationship between blood lead and IQ. As a result, the Chari *et al.* analysis does not support a need to modify the Pb NAAQS for protection of low-SES populations. At this time, the methods by which to consider non-chemical stressors in risk assessment are an active area of investigation and debate within the scientific community and among policy makers. The Chari *et al.* analysis does not inform this debate for the impact of SES on the relationship between blood lead and cognitive impacts. An appropriate analysis would need to focus, at a minimum, on a data set for the same blood lead metric, in children with blood lead measures and cognitive testing at approximately the same age.
